# Transcriptomic analysis-guided assessment of precision-cut tumor slices (PCTS) as an ex-vivo tool in cancer research

**DOI:** 10.1038/s41598-024-61684-1

**Published:** 2024-05-14

**Authors:** Sumita Trivedi, Caitlin Tilsed, Maria Liousia, Robert M. Brody, Karthik Rajasekaran, Sunil Singhal, Steven M. Albelda, Astero Klampatsa

**Affiliations:** 1 Division of Hematology and Oncology, Department of Medicine, University of North Carolina at Chapel Hill, Charlotte, NC USA; 2 Division of Pulmonary and Critical Care Medicine, Department of Medicine, Center for Cellular Immunology, University of Pennsylvania, Philadelphia, PA USA; 3 Department of Otorhinolaryngology-Head and Neck Surgery, University of Pennsylvania, Philadelphia, PA USA; 4 Division of Thoracic Surgery, Department of Surgery, University of Pennsylvania, Philadelphia, PA USA; 5 Division of Cancer Therapeutics, The Institute of Cancer Research, London, UK

**Keywords:** Precision-cut slices, Ex-vivo, Immune response, Transcriptomics, Mesothelioma, Tumor model, Biological models, Gene expression analysis, Biological techniques, Cancer, Immunology

## Abstract

With cancer immunotherapy and precision medicine dynamically evolving, there is greater need for pre-clinical models that can better replicate the intact tumor and its complex tumor microenvironment (TME). Precision-cut tumor slices (PCTS) have recently emerged as an ex vivo human tumor model, offering the opportunity to study individual patient responses to targeted therapies, including immunotherapies. However, little is known about the physiologic status of PCTS and how culture conditions alter gene expression. In this study, we generated PCTS from head and neck cancers (HNC) and mesothelioma tumors (Meso) and undertook transcriptomic analyses to understand the changes that occur in the timeframe between PCTS generation and up to 72 h (hrs) in culture. Our findings showed major changes occurring during the first 24 h culture period of PCTS, involving genes related to wound healing, extracellular matrix, hypoxia, and IFNγ-dependent pathways in both tumor types, as well as tumor-specific changes. Collectively, our data provides an insight into PCTS physiology, which should be taken into consideration when designing PCTS studies, especially in the context of immunology and immunotherapy.

## Introduction

Advancements in cancer therapy and precision medicine highlight the greater need for preclinical models that can recapitulate the complex interactions between tumor cells and the tumor microenvironment (TME) to identify optimal treatment strategies. Although cell culture, murine models, and patient-derived xenografts provide key information about tumor cell biology, they are unable to replicate the complex human TME and extracellular matrix in a single model^1–3^. Precision cut tumor slices (PCTS), which are generated by cutting thin, viable cross sections of fresh tumors, offer a unique approach to study human solid tumors within an architecturally intact microenvironment where spatial relationships are left largely intact^4,5^.

Although an appealing tool, PCTS utilization is subject to at least one major potential confounding issue that merits detailed consideration. The tumor tissue that is sliced into PCTS is subjected to several stressors, including: cold ischemia during harvesting in surgery, physical trauma of tissue slicing into PCTS, loss of blood flow, potential hypoxia, and maintenance under conditions optimized for cell culture. Without a good understanding of the effects of these stressors on the physiology and gene expression in PCTS, the interpretation of any changes induced by experimental manipulations studies will be difficult.

To date, most evaluations of the physiologic status of PCTS have relied on histological appearance to assess the duration of time they are able to maintain a normal morphology^6,7^. This approach, however, is rather insensitive and limited by the small number of parameters that can be examined. Histologic approaches are also insufficient to look at multiple physiologic and pathologic pathways. A much more robust approach to obtain a more extensive evaluation is to use transcriptomics, where changes in many thousands of genes and many key pathways can be simultaneously evaluated over time. Surprisingly, there is a relatively paucity of genomic data available, especially in tumors, and some of these data are contradictory. Bigaeva et al. provided a comprehensive characterization of the dynamic transcriptional changes in PCTS from normal and fibrotic mouse and human tissues using mRNA sequencing comparing fresh PCTS to those cultured for 48 h^8^. They demonstrated that explantation and culture were associated with extensive transcriptional changes and, interestingly, impacted PCTS in a relatively similar way across organs in both species by triggering an inflammatory response and fibrosis-related extracellular matrix (ECM) remodeling. In marked contrast, Ghaderi et al.^4^ did mRNA sequencing of formalin-fixed paraffin embedded (FFPE) tissue from five pancreatic ductal cancer samples at baseline and their matched PCTS cultured at 24, 48, and 72 h. The number of differentially expressed genes that they reported was extremely small, ranging from zero to only 56 genes.

The goal of this study was to examine the gene expression changes occurring in PCTS fresh human tumor samples over time. Our tumor data were consistent with those of Bigaeva et al. in normal tissues, finding extensive spontaneous changes at the transcriptomic level that tended to be most prominent in the first 24 h of culture with relative stabilization from 24 to 72 h^8^. These transcriptomic changes need to be considered when designing and analyzing PCTS experiments.

## Results

### HNC and Meso PCTS maintain normal tumor architecture and demonstrate up to 72 h viability in culture

**Figure 1:**
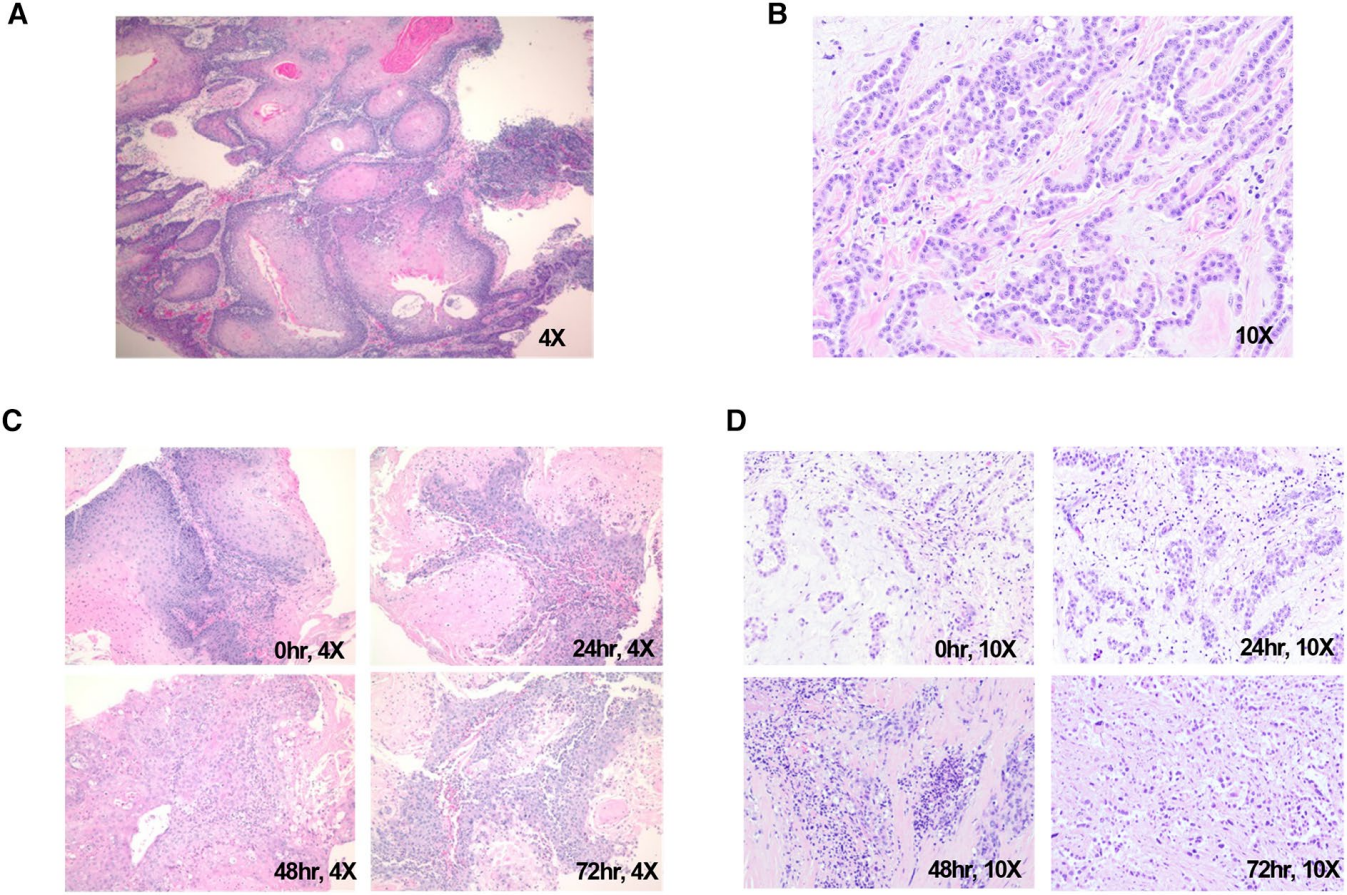
PCTS morphology and viability. H&E staining of fixed PCTS 3 μm sections showed that generation of PCTS using the Compresstome instrument retained intact tumor morphology in both HNC (**A**, 4X) and Meso tumors (**B**, 10X). PCTS stained every 24 h for 3 days showed that tumor architecture is preserved during the 72-h timeframe for both tumor types, with no signs of necrosis seen microscopically, suggesting the PCTS are viable (**C**, 4X images; D, 10X images).

H&E stains of FFPE sections from PCTS of both HNCs and Mesos demonstrated morphological characteristics that are typical of these cancers (Fig. 1A,B)*.* Over the course of a 72 h culture, PCTS maintained structural integrity without visible necrosis over time as shown in Fig. 1C,D respectively.

### PCTS undergo major culture-induced transcriptomic changes in the first 24h of culture

Transcriptomic analyses were conducted on PCTS from tumors from HNC (n = 3) and Meso (n = 3) patient samples. All tumors had at least one freshly cut PCTS that was immediately fixed (“Fresh”) and PCTS analyzed after 24 h in culture (“24 h”). In a subset of patients, data was also collected from PCTS at 48 and 72 h after culture.

Using unsupervised hierarchical clustering (Supplementary Fig. 1A) and principal component analysis (Supplementary Fig. 1B), we found that the Meso PCTS were tightly grouped. Two of the HNC PCTS were similar in phenotype, while one case (HNC 3) displayed a somewhat different gene expression profile. In all cases, the fresh and 24 h samples from each patient clustered together. We initially focused on the total number of transcriptomic changes that occurred over the first 24 h after culture (Fresh vs 24 h). Using the criteria described in the methods section, hundreds of genes showed changes (Supplementary Fig. 1C), with more genes downregulated than upregulated. The 25 most increased and 25 most decreased genes are listed in Suppl. Table 1.

### Pathway analysis of PCTS transcriptomic changes reveals upregulation of wound healing and ECM pathways and downregulation of TCR activation and IFN-gamma signaling

**Figure 2:**
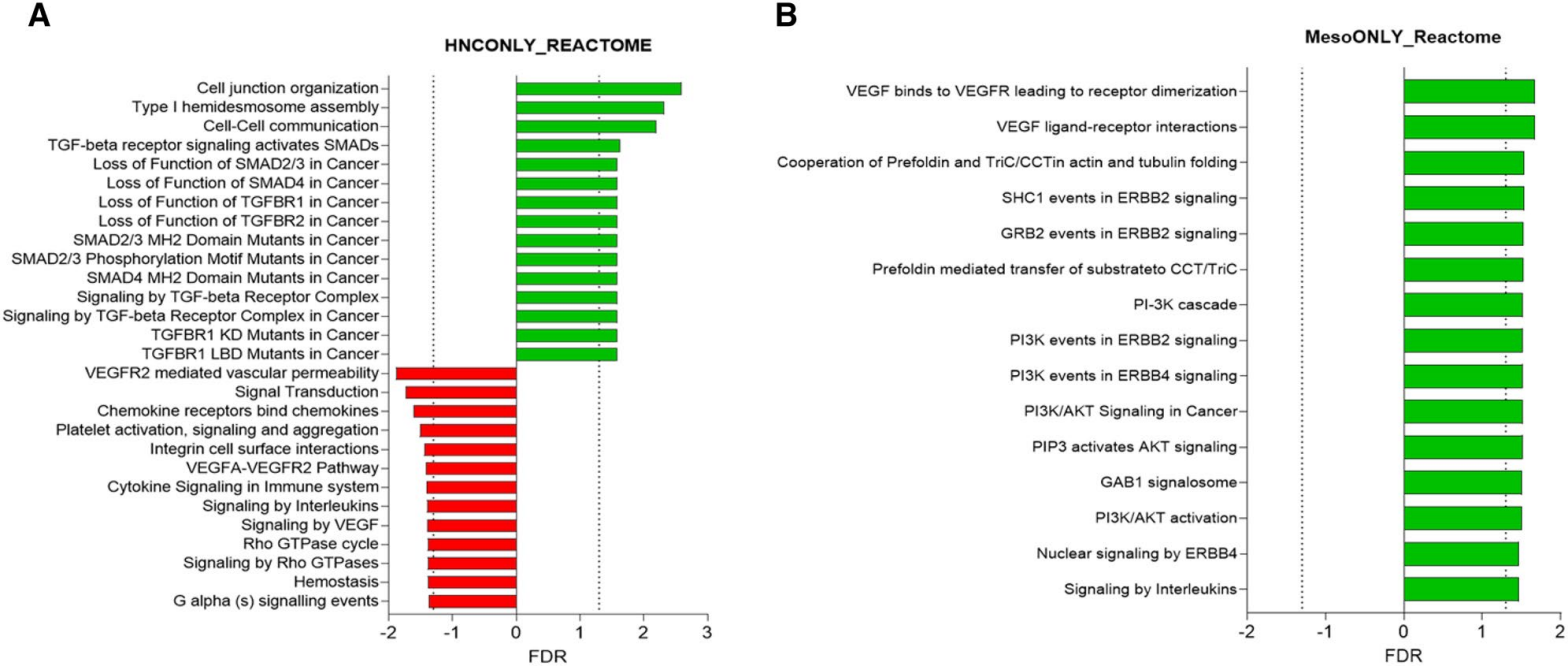
Pathways that changed in either HNC and Meso. Pathway analysis (Reactome) using lists of ranked genes that were changed (*p* < 0.05 and fold change > 2) in only the HNC PCTS (**A**) or only the Meso PCTS (**B**).

**Figure 3:**
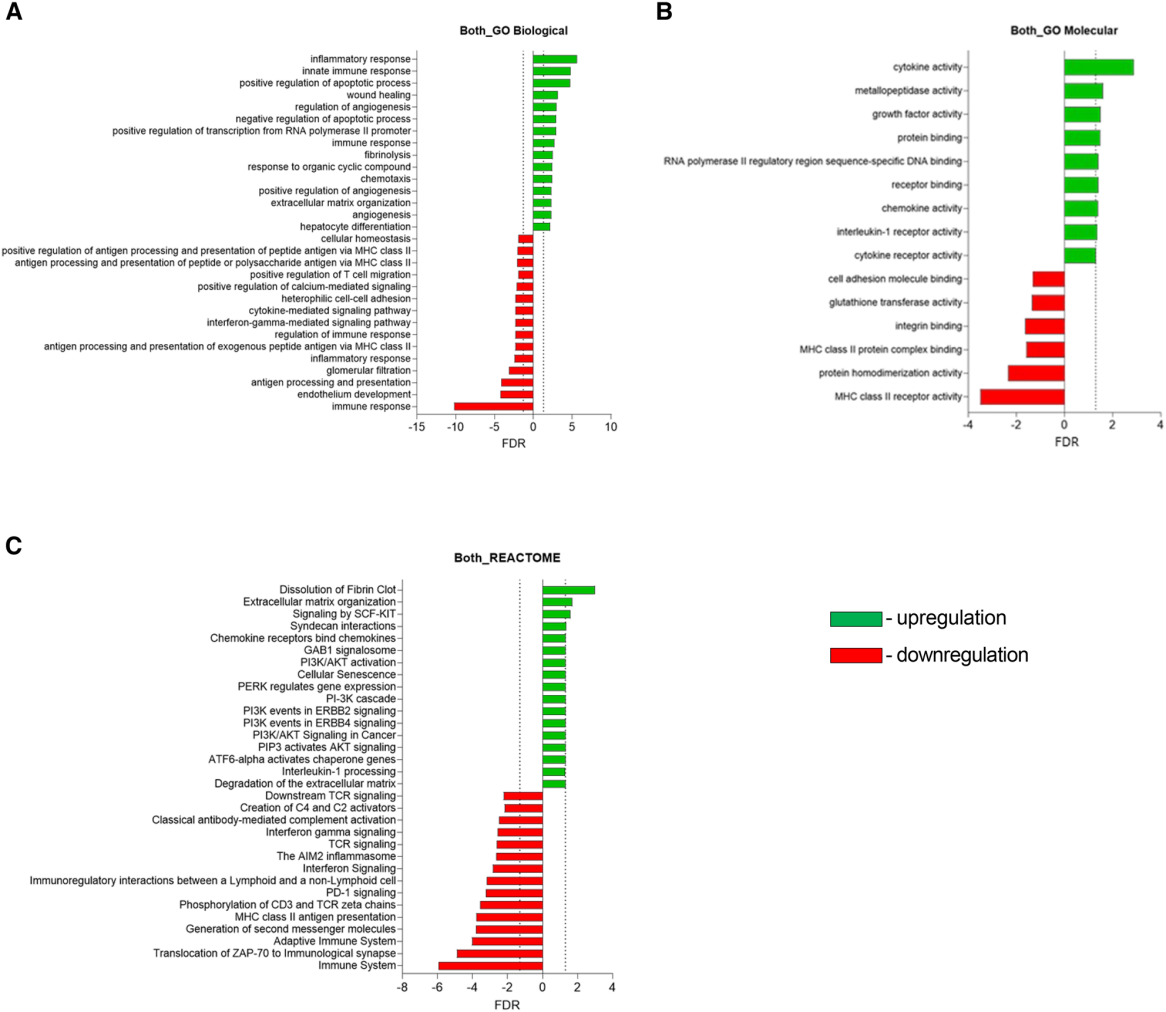
Pathways that changed in both HNC and Meso. Significantly changed genes were analyzed using the GO Biologic (**A**), GO Molecular (**B**), and Reactome Pathways (**C**).

We next conducted a pathway analysis of the changed genes. We noted that some genes were changed in both tumor types, but that there were also genes that appeared to change in the HNC but not in the Meso tumors and vice versa. In the HNC PCTS, increases in cell-to cell-junctional molecules and TGF-β and VEGF-related signaling pathways were seen, with downregulation in some chemokine/cytokine pathways. In the Meso PCTS, increases in VEGF and PI3K pathways were observed (Fig. 2). Our primary analyses performed on genes that were changed in both Meso and HNC revealed several significantly changed GO biological (Fig. 3A), GO molecular (Fig. 3B), and Reactome pathways (Fig. 3C). Of note were *upregulation* of pathways involved in extracellular matrix and fibrin reorganization and fibrinolysis, wound healing, angiogenesis, PI3K activation, and a subset of inflammatory and immune response genes. On the other hand, there were *downregulation* of pathways involved in TCR activation, IFNγ signaling, compliment activation, HLA Class 2 expression, and a different subset of inflammatory and immune response genes.

### Gene comparisons-housekeeping and tumor genes

**Figure 4:**
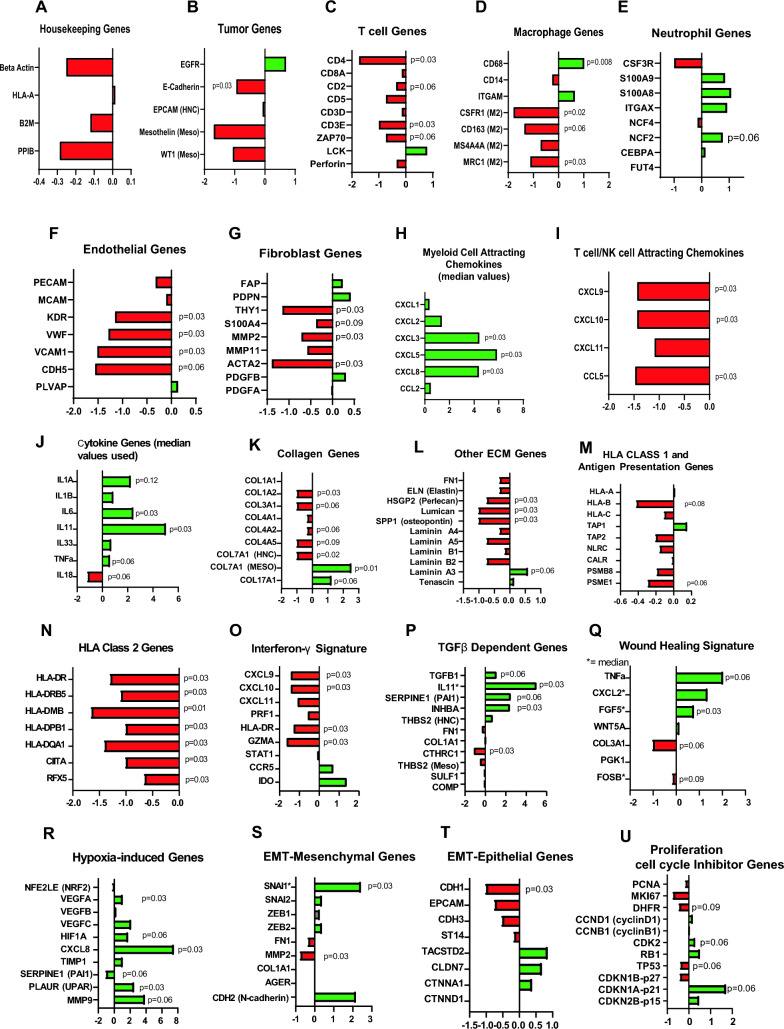
Summary of Gene Expression Changes. The log2 fold (X-axes) changes in mRNA expression levels at 24 h in a number of specific genes in key categories including (**A**) housekeeping genes, (**B**) tumor selective genes, (**C**) T cells genes, (**D**) macrophage genes, (**E**) neutrophil genes, (**F**) endothelial genes, (**G**) fibroblast genes, (**H**) myeloid attracting chemokine genes, (**I**) T cell/NK cell attracting chemokine genes, (**J**) cytokine genes, (**K**)collagen genes, (**L**) extracellular matrix proteins genes, (**M**) HLA Class 1 and antigen presentation genes, (**N**) HLA Class 2 genes, (**O**) Interferon gamma-induced genes, (**P**) TGFβ induced genes, (**Q**) Wound healing signature genes, (**R**) Hypoxia-induced genes, (**S**) EMT-mesenchymal genes, (**T**) EMT-epithelial genes, (**U**) Proliferation/Cell Cycle genes. Red = downregulated at 24 h, green = upregulated at 24 h. *p* values calculated by Wilcoxon test (*p* > 0.05). *p* values for significant or borderline significant changes are noted on the graphs.

Given these pathway changes and our interest in using the PCTS to study immunologic questions, we compared the fold changes in mRNA expression levels of Fresh versus 24 h PCTS in several key categories in Fig. 4. A set of housekeeping genes (Fig. 4A, Suppl. Table 2A and Suppl. Figure 2A) were all expressed at high levels, however, with a great deal of basal heterogeneity between and among tumor types. There was a slight trend toward downregulation of these genes at 24 h, but no statistically significant differences, suggesting no widespread changes in cell viability. A set of tumor selective genes (Fig. 4B, Suppl. Table 2B and Supplementary Fig. 2B) were expressed heterogeneously. As would be expected, expression levels of epithelial-related mRNAs, like EGFR and E-cadherin, were much higher in HNCs. There were little changes in these genes. In contrast, most of the tumor-related genes in the Meso PCTS were downregulated at 24 h, suggesting some loss of tumor cells.

### Gene comparisons- cell type selective genes

We next evaluated mRNAs that are characteristically expressed in specific cell types. The T cell selective genes were expressed with basal heterogeneity (Fig. 4C, Suppl. Table 2C and Suppl. Figure 2C). One of the Meso tumors was much “hotter” than the other PCTS where the expression levels were rather low (Supplementary Fig. 2C). There was a trend towards decrease in T cell genes (8 of 9 mRNAs were decreased), but most of the changes were not significant. The degree of downregulation of T cell genes in the HNCs in the 24 h PCTS was greater than seen in the Meso PCTS. Macrophage selective genes (Fig. 4D, Supplementary Table 2D, Supplementary Fig. 2D) showed a great deal of basal heterogeneity, but the expression levels were higher in Meso PCTS. The general macrophage marker (CD68) was significantly increased at 24 h, but CD14 and ITGAM were not. However, the M2 marker genes (CSFR1, CD163, MS4A4A, and MRC1) were all decreased, suggesting a shift to a more M1-like phenotype. No significant changes were noted in neutrophil genes (Fig. 4E, Supplementary Table 2E and Supplementary Fig. 2E). There was heterogeneity in endothelial selective genes (Fig. 4F, Supplementary Table 2F and Supplementary Fig. 2F). Most endothelial cell genes were downregulated in the 24 h PCTS, with the changes being significant or near significant. As expected, basal expression of fibroblast selective genes were higher in the Meso compared to the HNC PCTS (Fig. 4G, Supplementary Table 2G and Supplementary Fig. 2G). Most of the fibroblast genes were downregulated with changes in THY1, MMP2, and ACTA2 being significant.

### Gene comparisons-chemokine and cytokine genes

Levels of chemokines known to attract myeloid cells were first examined (Fig. 4H, Supplementary Table 3A, Supplementary Fig. 3A). Basal levels of CCL2 were much higher in the Meso vs HNC PCTS. Compared to baseline, expression of all myeloid chemokine mRNAs was higher in the 24 h PCTS and among the largest increases we observed in any gene set. The fold increases were higher in the Meso compared to HNC PCTS. In contrast, expression of the lymphoid-attracting chemokine mRNAs examined were much lower in the 24 h PCTS (Fig. 4I, Supplementary Table 3B and Supplementary Fig. 3B). We observed statistically significant and large decreases in expression in the CXCL9, CXCL10, and CCL5 genes. The decreases in expression were greater in the HNC compared with Meso PCTS. There was some heterogeneity in the basal levels, but in general, these chemokines were higher in the Meso tumors. Most cytokine mRNAs examined showed relatively few changes (Fig. 4J, Supplementary Table 3C and Supplementary Fig. 3C). However, IL-6 and IL-11 showed large and significant increases at 24 h. IL-1A, IL-1B, and IL-33 showed increases that did not reach statistical significance. TNFα and IFNγ mRNAs were present at only low levels.

### Gene comparisons-extracellular matrix (ECM) genes

Given the results of our pathway analysis above showing changes in ECM gene expression, we examined the expression levels of collagen mRNAs and other ECM mRNAs (Fig. 4K, Supplementary Table 3D, and Supplementary Fig. 3D). There was tumor-specific basal heterogeneity, but we observed a consistent down-regulation of Collagen 1, 3, and 4 mRNAs. Regarding other ECM genes, there was some tumor-specific basal heterogeneity (Fig. 4L, Supplementary Table 3E, and Supplementary Fig. 3E), but most were decreased at 24 h, with more marked decreases in the Meso vs HNC PCTS.

### Gene comparisons- HLA class 1, class 2, and antigen presentation machinery (APM)

HLA-Class 1 genes, B2M, and APM genes were expressed at very different levels among the PCTS (Fig. 4M, Supplementary Table 4A, Supplementary Fig. 4A). There was a slight trend toward downregulation of the HLA Class I genes and antigen presenting machinery mRNAs in HNC compared with Meso, but no significant changes were seen. In contrast, there was a very consistent and strong downregulation in the mRNAs of HLA Class II genes and Class II-regulating transcription factors, with more downregulation in HNC vs Meso PCTS (Fig. 4N, Supplementary Table 4B, Supplementary Fig. 4B).

### Gene comparisons-IFNγ signature

A large amount of heterogeneity in the basal levels of mRNAs of genes induced by IFNγ was seen (Fig. 4O, Supplementary Table 4C, Supplementary Fig. 4C), but most genes were down regulated (4 significantly), except for IDO. The decreases in expression were greater in the HNC vs Meso PCTS.

### Gene comparisons-TGF-β-induced genes

No clear pattern was evident in a panel of well described TGF-β-induced genes. Aside from TGF-β1 itself, all other mRNAs of TGF-β1 genes were higher in Meso PCTS. IL-11, SERPINE1, and INHBA mRNAs were significantly increased, however, most other genes decreased or were unchanged (Fig. 4P, Suppl. Table 4D, Supplementary Fig. 4D).

### Gene comparisons-wound healing genes

Genes from a wound healing signature described by Vitali et al. were examined (Fig. 4Q, Suppl. Table 5B, Supplementary Fig. 5B)^9^. There was tumor-specific basal heterogeneity, but there was a trend towards an increase in the wound signature mRNAs especially in the Meso PCTS; all but COL3A1 genes were increased, but only FGF5 reached significance.

### Gene comparisons-hypoxia-induced genes

All the hypoxia genes examined showed large and mostly significant increases at 24 h (Fig. 4R, Suppl. Table 5A, Suppl Fig. 5A), apart from NFE2L2 (NRF1). The hypoxic response was generally greater in the Meso PCTS.

### Gene comparisons-epithelial to mesenchymal transition (EMT) genes

Sets of genes that define a mesenchymal phenotype (Fig. 4S, Suppl. Table 5C, Supplementary Fig. 5C) or epithelial phenotype (Fig. 4T, Suppl. Table 5D, Supplementary Fig. 5D) were compared. As expected, the mesenchymal genes were higher at baseline in Meso versus HNC tumors (since Meso is a more mesenchymal tumor), but there was no clear trend toward increased mesenchymal gene expression. In contrast, basal levels of many epithelial genes were much higher in HNC PCTS than meso PCTS. Except for CDH1, there was no significant downregulation of epithelial genes, however.

### Gene comparisons-proliferation-related genes and cell cycle inhibitor genes

There were no clear changes in the proliferation-related mRNAs (Fig. 4U, Suppl. Table 6A, Supplementary Fig. 5E), with a slight trend toward decreased proliferation gene expression seen in the HNC PCTS. Changes in cell cycle inhibitor mRNAs were mixed: p21 and p15 increased, but p53 and p27 decreased (Fig. 4U, Suppl. Table 6B, Supplementary Fig. 5E).

### Gene expression changes over time mostly occur in the first 24 h post-slicing

**Figure 5:**
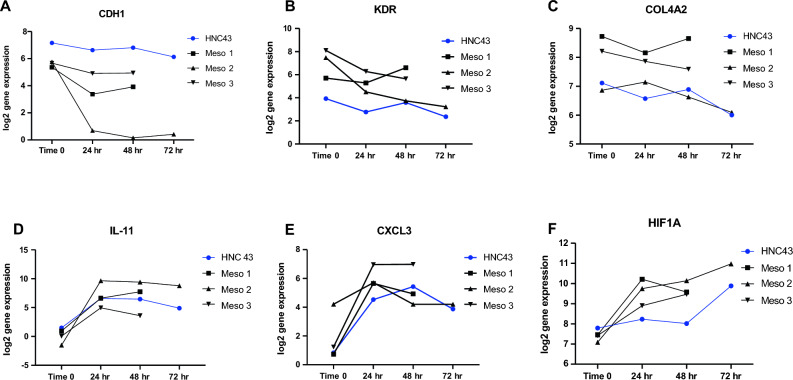
Gene Expression Changes over Time. The log2 gene expression values in 4 tumor PCTS (3 Meso and 1 HNC) were measured at baseline (time 0), and at 24, 48, and 72 h after culture. Examples of genes downregulated include (**A**) E-cadherin (CDH1), (**B**) VEGFR2 (KDR), and (**C**) collagen4A2. Examples of genes upregulated include (**D**) Interleukin 11 (IL-11), E) CXCL3, and (**F**) HIF1A.

In general, the largest changes in gene expression were seen between the fresh and the 24 h PCTS. Values then tended to plateau, with the most stable period between 24 and 48 h. Examples of this pattern for three downregulated genes are shown in Fig. 5, E-cadherin (CDH1) (Fig. 5A), KDR (Fig. 5B), and COL4A2 (Fig. 5C). Examples of this pattern for three upregulated genes are IL-11 (Fig. 5D), CXCL3 (Fig. 5E), and HIF1A (Fig. 5F). Supplemental Fig. 6 depicts the mRNA expression changes over time of representative housekeeping (6A), tumor (6B), T cell-selective (6C), macrophage-selective (6D), endothelial-selective 6E), fibroblast selective genes (6F), cytokine (6G), myeloid-attracting chemokine (6H), lymphocyte attracting chemokine (6I), extracellular matrix (6J), hypoxia (6K) or miscellaneous (6L) mRNAs.

## Discussion

Recent advances in cancer research have expanded treatment options for many cancers, yet their use remains limited to select populations^10,11^. Thus, there is a need for predictive preclinical models with which we can understand translational failures and accelerate the development of novel therapeutics. Historically, 2D and 3D cell cultures, murine and other animal models, as well as patient-derived xenografts, have been utilized for preclinical studies, however, they are limited by their inability to fully recapitulate the complex human TME. PCTS are an ex vivo system that largely preserves the 3D tumor architecture, along with its TME and stroma. They are relatively easy to produce, and they can offer a platform for conducting therapy-based experiments on human solid tumor tissue^5^. However, our current understanding of the complex changes that occur during slice production and culture (even without any treatment-induced changers) is quite limited. In this study, we used transcriptomics to comprehensively study the mRNA changes that occur in two tumor types, namely HNC and Meso, especially within the first 24 h following slicing (i.e. PCTS production).

Our data showed that PCTS from HNC and Meso retained histological fidelity for up to 72 h in culture. Other studies using PCTS derived from HNCs have demonstrated a similar viability of 2–7 days^6,7,12^. This is likely tumor-type dependent, as others have reported that PCTS from other tumor types (murine tumors, colorectal, non-small cell lung cancer and liver tumors) were viable for up to 12 days^8,13–15^. It may also be culture-specific since, in our experience, different culture media, with or without serum supplementation, and different culture plates can affect PCTS viability^12^. Collectively, these data demonstrate the importance of (1) assessing viability for each type of tumor studied and (2) fully optimizing the culture conditions employed.

The major finding of this study is that, although the PCTS appear to remain relatively unchanged histologically in the first 72 h of culture, they undergo major transcriptomic changes during this period, especially during the first 24 h following slicing. Unlike the study of Ghaderi et al.^4^ which reported very few changes (zero to only 56 genes) in PCTS after culture, our tumor data are similar to the results published by Bigaeva et al.^8^ that demonstrated extensive transcriptional changes from PCTS made from normal and fibrotic mouse and human tissues.

We observed that the expression of some cell type-specific mRNAs decreased. This included mRNAs associated with T cells (especially the mRNA for CD4), fibroblasts, and most prominently, endothelial cells. Interestingly, although major macrophage-specific mRNAs (CD68, CD14, and ITGAM) were relatively unchanged, the mRNAs associated with an M2 phenotype (CSFR1, CD163, MS4A4A, and CRC1) were decreased, suggesting a change toward a more M1, anti-tumor status.

One of the major changes we observed was in the chemokine/cytokine milieu of the PCTS. IFNγ-related mRNAs were consistently downregulated, including the mRNAs for chemokines that attract lymphocytes (such as CXCL9, 10, 11 and CCL5) and the HLA Class II-regulating mRNAs (i.e., CIITA and RFX5). Expression of HLA Class I and antigen-presenting machinery mRNAs were less affected. By contrast, mRNAs for chemokines that attract myeloid cells (such as CXCL2, CXCL5, CXCL8, and CCL2), were markedly and significantly upregulated. In addition, several inflammatory cytokine gene mRNAs were highly increased, including IL-1A, IL-1B, TNF, and IL-6. One of the most highly upregulated mRNAs was IL-11, which was also prominently increased in the study examining PCTS of normal tissues^8,16^. IL-11 is a member of the IL-6 family of cytokines and has been implicated in the pathogenesis of fibrosis and solid malignancy, as well as inflammation^17,18^.

Another prominent set of changes that we observed involve the upregulation of mRNAs regulating tissue injury and repair, including the wound-induced matrix protein tenascin. This initial response may be the result of the traumatic effects of slicing the PCTS and was also observed in the normal tissue PCTS^8^. However, many ECM mRNAs were downregulated, including many collagen genes, as well as the extracellular matrix proteins, Perlecan and Lumican, especially in the Meso PCTS. Consistent with matrix remodeling, mRNA’s for MMPs 1, 3, 9, and 10 were markedly upregulated. TGF-β1 mRNA and several TGF-β stimulated genes (IL-11, Serpine 1, INHBA) were significantly increased. Finally, there was transcriptomic evidence of a hypoxic response, with many hypoxia-associated mRNAs (VEGFA, VEGFC, HIF1a, CXCL8, PLAUR, and MMP3) showing significant or close to significant upregulation.

Overall, our transcriptomic analysis data in tumors agrees with that previously seen in normal tissues^8^ and suggest that, when tumors are removed from the body, sliced into PCTS, and cultured ex vivo for 24 h, they undergo a complex set of changes. There is a tendency toward loss of endothelial cells, fibroblasts, and lymphocytes, and a shift towards an M1 vs M2 macrophage phenotype. This is associated with a downregulation of IFNγ-induced mRNAs and processes. The other prominent changes relate to wounding and hypoxia responses that are highlighted by marked changes in the cytokine/chemokine milieu and extracellular matrix. These wounding and hypoxic programs likely begin the same way as would be observed in vivo but are altered in the PCTS because the normal influx of blood cells (i.e. platelets, neutrophils, monocytes, fibrocytes, etc.) and angiogenesis, cannot occur due to lack any vascular connections. The largest changes seemed to occur in the initial 24 h of PCTS culture and then tended to stabilize. This is of importance as it suggests that experimental manipulations to the PCTS would be best be studied 24 h after slicing to allow the PCTS to “de-stress”, adapt to the culture conditions and mRNA levels to stabilize.

Given these rather substantial changes, each investigator will need to account for how these changes might affect specific experiments and consider validating these changes at the protein level. Our findings suggest that studies looking at effects of manipulations on the tumor cells themselves (i.e., via addition of chemotherapy or drugs), might be relatively unaffected^19–21^, but are likely tumor cell specific. In our examples, tumor-selective mRNAs in the HNCs (EGFR, CDH1) were stable, however, mesothelioma-selective mRNAs (i.e., mesothelin and WT1) were reduced. Studies requiring antigen presentation might be misleading. It is unclear how experiments in which cells are added to PCTS to monitor migration would be affected^21–23^, because of the changes occurring in the extracellular matrix. In the handful of studies that have utilized PCTS for CAR-T cell or genetically-engineered macrophage assessment, results have been reported to show targeted tumor cell death with associated cytokine release in the supernatant^23,24^, with the immune responses seen in the macrophage study shown to be consistent between patient-derived PCTS^23^. In our opinion, the utility of PCTS as a platform in adoptive cell transfer research is promising but requires further investigation.

There are a number of limitations to our study that should be considered. We studied only two types of tumors, each showing its own specific changes. Although most changes that we observed were similar between the two tumor types, basal levels of many mRNAs were quite different amongst the tumors. Some changes occurred in the Meso PCTS, but not the HNC PCTS, and vice versa, we also saw examples of changes in opposite directions. For example, after culture, COL7A1 mRNA was significantly decreased in HNC, but significantly increased in Meso. Accordingly, each tumor type should be carefully studied for specific changes. Another limitation is that our transcriptomic data was derived from only a small number of tumors and lacked multiple biological replicates at all time points. Because of this, our primary method of analysis was to use each tumor as its own control and calculate fold changes over time rather than comparing the mean values of all PCTS at different time points. However, despite having a relatively low level of statistical power, many of the changes observed were consistent and significant using paired t-tests. The validity of our conclusions is bolstered by the somewhat surprising similarities between the upregulated mRNAs that we observed in our tumor slices compared to those reported previously in normal tissues^8^. Remarkably, all the 6 most upregulated mRNAs increased in the PCTS from normal tissues (IL-6, CXCL5, CXCL8, MMP1, MMP3, and TFPI2) were among our top 36 most upregulated genes and had an average fold increase of 24.4. However, additional transcriptomic data to define changes in PCTS over time would benefit the field. Finally, this study focused only on generating transcriptomic data. Given the potential lack of correlation between mRNA and protein expression, especially in tumors, it would be of value to validate relevant transcriptomic findings using proteomic methods such as immunoblotting, immunohistochemistry and/or flow cytometry. Preliminary studies by our group suggest that it is possible to obtain enough cells to conduct multi-color flow cytometry from individual slices.

In summary, the ability of PCTS to retain the original tumor microenvironment and architecture makes them an attractive model to study tumor biology, therapies, immunology and immunotherapies. However, the investigator should be aware that there are multiple dynamic transcriptional changes that occur after slicing and during the early culturing of PCTS that can differ among tumor types and among patients. Our findings indicate that this complex program of changes involves the potential decrease of several cell types within the PCTS, along with wounding and hypoxia responses that are highlighted by marked changes in the mRNA levels of cytokines, chemokines, HLA Class II molecules, and extracellular matrix. Depending on the experimental questions asked, these “baseline” changes need to be carefully considered.

## Materials and methods

### Tissue samples

Surplus resected tumor material from human head and neck (HNC) and mesothelioma (Meso) tumors were obtained from the operating room immediately after surgical resection. Informed written consent related to two IRB approvals (Penn IRB protocols #813004 and #417200) were obtained prior to donation. Samples were transported from the operating room to the laboratory in ice cold media (DMEM/F12, 10% FBS, 1% Penicillin/Streptavidin) and processed within 1 h. The tissue from HNC (n = 3) and Meso (n = 3) tumors were used for transcriptomic evaluation.

### Preparation of precision cut tissue PCTS

PCTS of 300 μm in thickness were prepared using a Compresstome (Precisionary Instruments LLC, VF-300). Tumor samples were mounted on the tissue plunger and embedded in 2% low-melt agarose, as previously described^5^. Several PCTS were placed immediately in 10% formalin for fixation and labeled “Fresh/Time 0/0 h”. The remaining PCTS were placed on top of cell culture inserts (Millipore) in 24-well tissue culture plate and cultured in DMEM/F12 media supplemented with 10% FBS and 1% penicillin/streptomycin at 37 °C in a 5% CO_2_ incubator. At various time points (24, 48, and 72 h), the PCTS were fixed in 10% formalin. All fixed PCTS were subsequently embedded in paraffin and standard 3 μm sections were cut and placed on glass slides. Slides were used for H&E or transcriptomic analyses.

### HTG transcriptome panel

FFPE slides were reviewed, and areas of tumor marked by a head and neck pathologist. Depending on the size, one to three marked slides from each PCTS were sent to HTG Molecular Diagnostics at the HTG VERI/O commercial laboratory in Tucson, Arizona. For this study, we used a newly available HTG Transcriptome Panel that interrogated 19,398 genes simultaneously (https://www.htgmolecular.com/assays/htp). HTG EdgeSeq probes target a single location of each RNA transcript resulting in a single probe sequence per RNA transcript. The counts therefore are stoichiometrically equal to the number of transcripts in the sample and normalization by transcript length is not required. The HTG EdgeSeq data is transformed into gene counts per million counts (CPM) and normalized CPM gene reads were provided by HTG. Genes with low expression levels (less than 1 CPM) were filtered leaving approximately 11,000 genes for analysis.

### Differentially expressed gene analysis

Given our goal of examining changes in the PCTS over time, the relatively small number of PCTS examined, the known differences between HNC and Meso, the presumed heterogeneity between individual tumors of even the same histology, and the lack of biological replicates at many of our time points, our primary method of analysis was to use each baseline (Time 0) PCTS as the control for comparison to later time points from the same tumor, rather than comparing the averages of groups of PCTS at different time points. This allowed us to use paired analyses and gave us six comparisons at each time point for each gene to analyze. For each of the six tumors, we calculated the fold change of any given gene at 24 h compared to time zero and calculated the median fold change. We then screened for genes that had a paired t test value of < 0.05 (uncorrected) and looked at various fold-change thresholds.

Due to our relatively small sample sizes, we generated lists of changed genes for pathway analysis using the following criteria (with a bias towards being inclusive). All genes with an average greater than twofold change from the Time 0 PCTS to the 24 h PCTS were reviewed manually. If the p value was > 0.05, we reviewed for outliers. If consistent changes were seen except for one outlier, the gene was kept in the list. If there were no clear outliers, the gene was cut from the list. Our primary analysis was performed on genes that were changed in both lung mesothelioma and head and neck cancer.

We also conducted a pathway analysis of the differentially expressed genes. For pathway analysis and Gene Ontology analysis, up- and down-regulated DEG were uploaded to InnateDB^25^. InnateDB looks for over representation of DEG within the KEGG, REACTOME and Gene Ontology databases. p values were adjusted for multiple comparisons using the B–H method and an FDR < 0.05 considered significant^26^.

### Supplementary Information

Supplementary Information.

## Data Availability

Gene expression data were deposited into the Gene Expression Omnibus database under accession number GSE250038. To review GEO accession GSE250038, go to: https://www.ncbi.nlm.nih.gov/geo/query/acc.cgi?acc=GSE250038. Token: mhadwayyvzupvmd.
