# Supplementary material for: Transcriptomic analysis-guided assessment of precision-cut tumor slices (PCTS) as an ex-vivo tool in cancer research
Source: Sci Rep. 2024 May 14;14:11006. doi: 10.1038/s41598-024-61684-1 (PMC11094020; doi:10.1038/s41598-024-61684-1)
Supplement: Supplementary file 1 — Supplementary Information. [file 41598_2024_61684_MOESM1_ESM.pdf]

Transcriptomic analysis-guided assessment of precision-cut tumor slices (PCTS) as an ex-vivo tool in cancer research

Sumita Trivedi[1], Caitlin Tilsed[2], Maria Liousa[2], Robert M Brody[3], Karthik Rajasekaran[3], Sunil Singhal[4], Steven M. Albelda[2], Astero Klampatsa[5*]

[1]Division of Hematology and Oncology, Department of Medicine, University of North Carolina, North Carolina, USA
[2]Center for Cellular Immunology and Division of Pulmonary and Critical Care Medicine, Department of Medicine, University of Pennsylvania, Philadelphia USA
[3]Department of Otorhinolaryngology-Head & Neck Surgery, University of Pennsylvania, Philadelphia, Pennsylvania, USA
[4]Division of Thoracic Surgery, Department of Surgery, University of Pennsylvania, Philadelphia, Pennsylvania, USA
[5]Division of Cancer Therapeutics, The Institute of Cancer Research, London, UK

A.

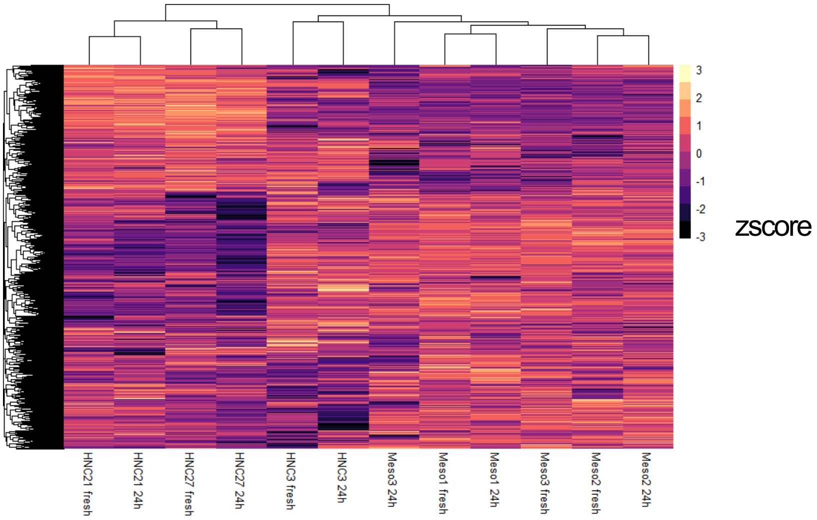

B.

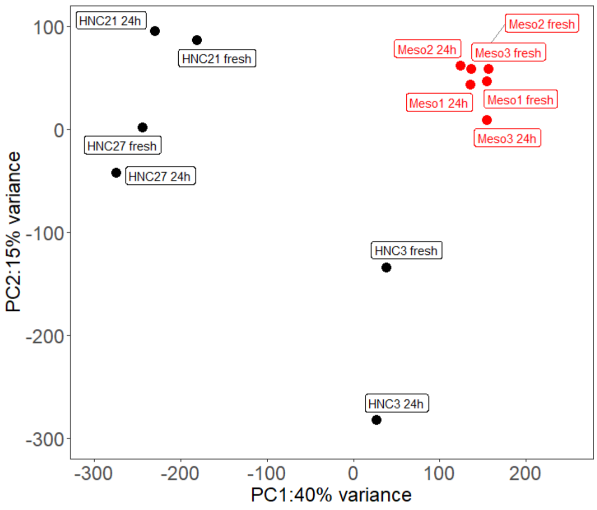

C.

Fold Change	Gene No
> 3-fold increase	52
> 2-fold increase	145
> 2-fold decrease	407
> 3-fold decrease	188
> 4-fold decrease	106

Suppl Figure 1. Transcriptomic analyses were conducted on PCTS from tumors from three HNC patients and three Meso patients at the time of harvest (time 0) and 24 hours later. Analyses included: A) unsupervised hierarchical clustering and B) principal component analysis. C) The table represents the number of genes changed at 24 hours using various thresholds.

Top Upregulated mRNAs	Top Downregulated mRNAs
ANGPTL4	ADH1C
C6orf141	ADORA3
CXCL3	AIF1
CXCL8	C5
FOSL1	CCL14
GPAT3	CCR2
HMGA2	CDH5
IL13RA2	F13A1
IL1A	FMO1
IL1RL1	FOSB
IL6	GGTA1
KIAA1549L	GJA5
MAFA	LST1
MMP1	MNDA
MMP10	MS4A6A
MMP3	OMD
NRIP3	P2RY13
PAQR5	PLA2G2A
RGS20	PLD4
RPA1	RAET1E
SERPINB2	RTN1
SH2D5	SIGLEC1
SMCO2	TLR7
TBL3	VSIG4
TNFRSF10D	ZBED8

Suppl Table 1. Top 25 upregulated and top 25 downregulated mRNAs. All mRNAs showed more than a 3-fold change (Fresh vs 24 hours) and had a $p<0.05$ on a paired t test.

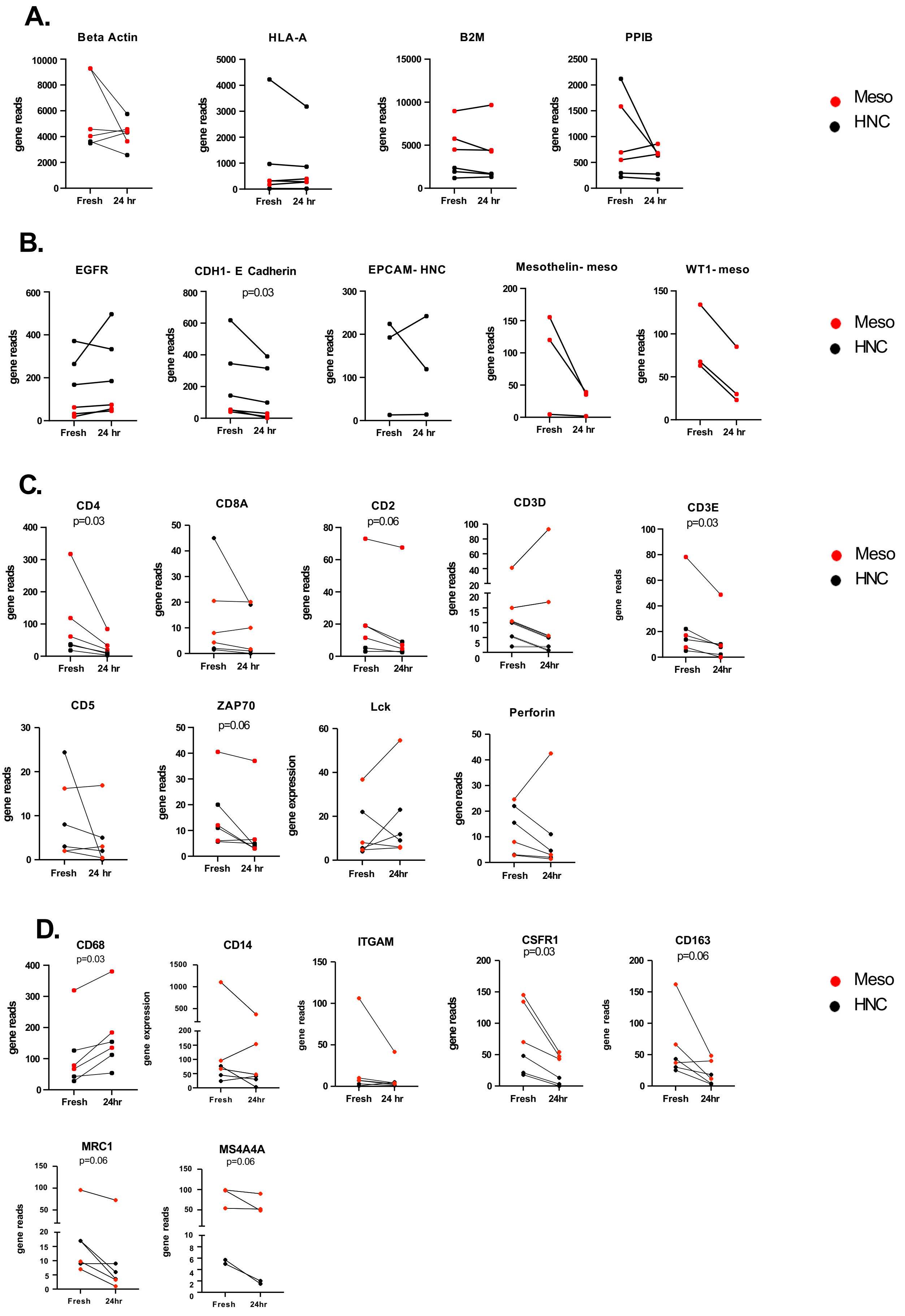
A.
Beta Actin
HLA-A
B2M
PPIB
gene reads
Fresh
24 hr
Meso
HNC
B.
EGFR
CDH1- E Cadherin
p=0.03
EPCAM- HNC
Mesothelin- meso
WT1- meso
C.
CD4
p=0.03
CD8A
CD2
p=0.06
CD3D
CD3E
p=0.03
24hr
CD5
ZAP70
p=0.06
Lck
gene expression
Perforin
D.
CD68
p=0.03
CD14
ITGAM
CSFR1
p=0.03
CD163
p=0.06
MRC1
p=0.06
MS4A4A
p=0.06

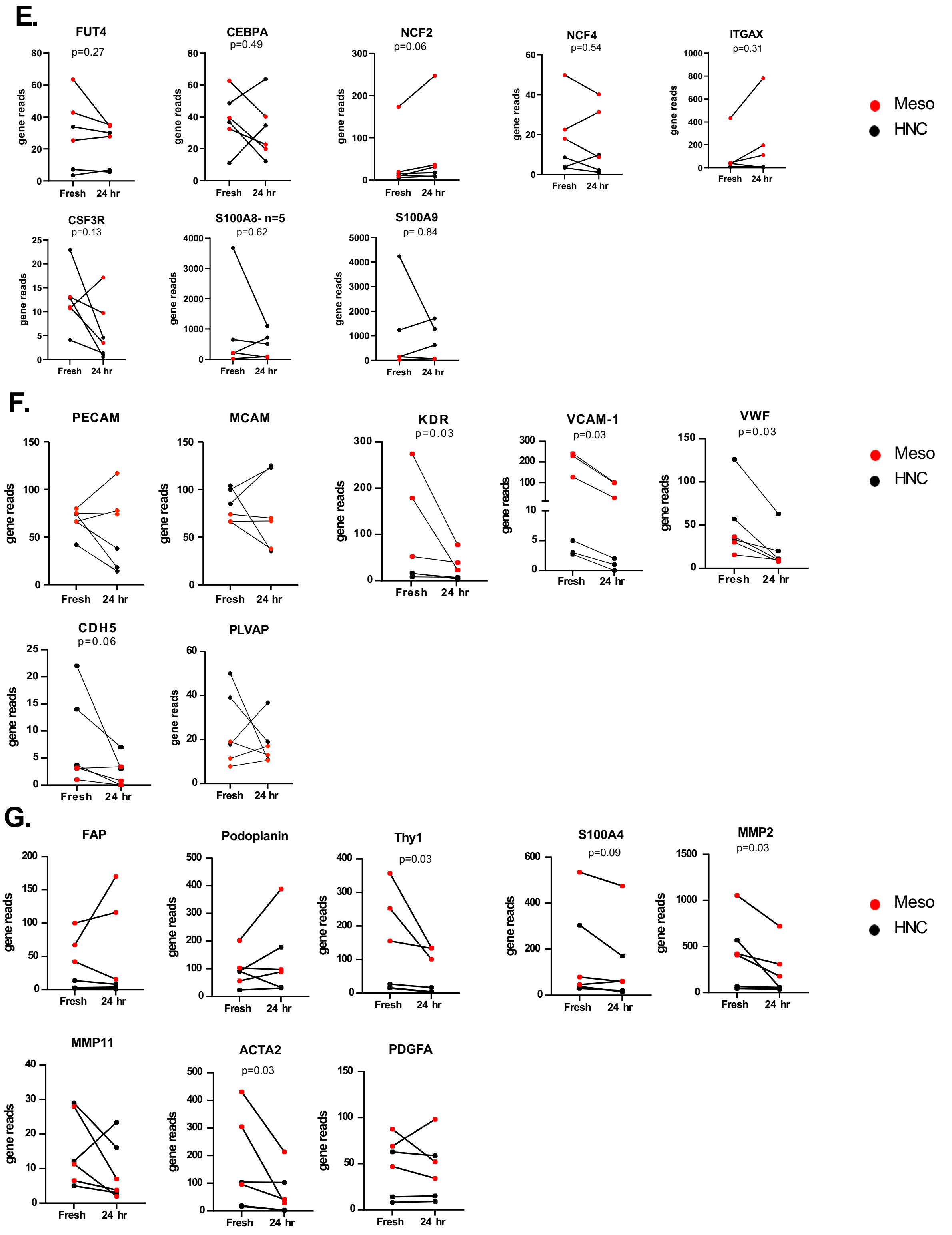

Suppl Figure 2. Gene Expression Comparisons between HNC and Mesothelioma. The mRNA expression levels (base 10) at baseline and at 24 hours for each Meso (red dots) and HNC (black dots) PCTS are plotted for several specific genes in key categories including: (A) housekeeping genes; (B) tumor selective genes; (C) T cell selective genes; (D) Macrophage selective genes; (E) Neutrophil selective genes, (F) endothelial selective genes; (G) fibroblast cell selective genes. Paired t-test p values are listed when significant or borderline significant (p< 0.05). (HNC: n= 3; Meso: n= 3)

Gene	Ave fold change (HNC)	Ave fold change (Meso)	Ave fold change (all)	p value (all) Wilcoxon ranked sum test
	**A. Housekeeping genes**			
Beta Actin	0.86	0.83	0.84	0.31
HLA-A	0.79	1.24	1.01	0.56
B2M	0.9	0.94	0.92	0.56
PPIB	0.68	0.96	0.82	0.56
	**B. Tumor-specific genes**			
EGFR	1.29	1.86	1.58	0.31
E-cadherin	0.75	0.29	0.52	0.03
EPCAM	0.95	---	---	0.99
Mesothelin	---	0.31	---	0.25
WT1	---	0.48	---	0.25
	**C. T cell genes**			
CD4	0.37	0.9	0.7	0.03
CD8A	0.48	1.2	0.7	0.21
CD2	0.57	0.6	0.6	0.06
CD5	0.45	0.8	0.6	0.19
CD3D	0.52	1.3	0.90	0.81
CD3E	0.53	0.4	0.5	0.03
ZAP70	0.43	0.8	0.6	0.06
Lck	2.57	1.2	1.9	0.56
Perforin	0.42	0.9	0.6	0.43
	**D. Macrophage genes**			
CD68	2.18	1.85	2.02	0.03
CD14	0.79	0.88	0.84	0.43
ITGAM	2.70	0.42	1.56	0.15
CSFR1	0.14	0.45	0.29	0.03
CD163	0.26	0.52	0.39	0.06
MS4A4A	0.35	0.79	0.61	0.06
MRC1 (CD206)	0.50	0.42	0.46	0.06
	**E. Neutrophil genes**			
FUT4	1.20	0.82	1.01	0.27
CEBPA	1.60	0.62	1.11	0.49
NCF2	1.23	2.17	1.70	0.06
NCF4	1.05	0.90	0.97	0.54
ITGAX	0.65	3.31	1.98	0.31
CSF3R	0.19	1.76	0.54	0.15
S100A8	1.58	3.60	2.02	0.69
S100A9	1.83	1.76	1.83	0.84
	**F. Endothelial cell genes**			
PECAM	0.39	1.21	0.80	0.56
KDR	0.50	0.39	0.45	0.03
VWF	0.43	0.39	0.45	0.06
VCAM-1	0.19	0.35	0.27	0.03
CDH5	0.20	0.48	0.34	0.06
PLVAP	0.92	1.18	1.05	0.56
	**G. Fibroblast genes**			
FAP	0.92	1.36	1.18	0.88
PDPN	1.20	1.49	1.34	0.31
THY1	0.35	0.55	0.45	0.03
S100A4	0.55	0.99	0.77	0.09
MMP2	0.59	0.62	0.61	0.03
MMP11	1.00	0.34	0.67	0.22
ACTA2	0.41	0.35	0.38	0.03
PDGFA	1.04	0.91	0.98	0.65

Suppl Table 2. Housekeeping, Tumor, and Cell Type Specific mRNA Expression Changes

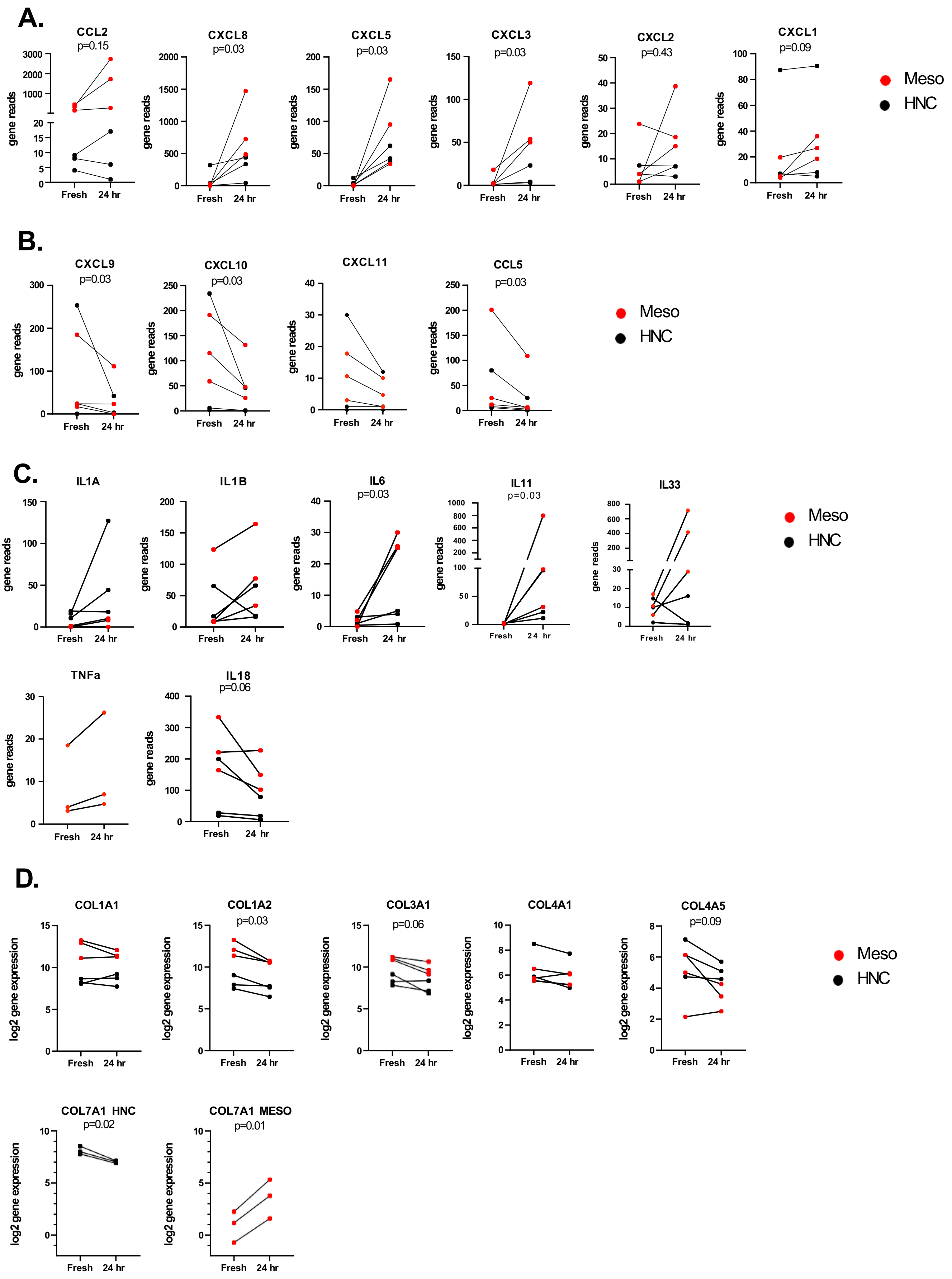
A.
CCL2
p=0.15
CXCL8
p=0.03
CXCL5
p=0.03
CXCL3
p=0.03
CXCL2
p=0.43
CXCL1
p=0.09
gene reads
Fresh
24 hr
Meso
HNC
B.
CXCL9
p=0.03
CXCL10
p=0.03
CXCL11
CCL5
p=0.03
gene reads
Fresh
24 hr
Meso
HNC
C.
IL1A
IL1B
IL6
p=0.03
IL11
p=0.03
IL33
TNFa
IL18
p=0.06
gene reads
Fresh
24 hr
Meso
HNC
D.
COL1A1
COL1A2
p=0.03
COL3A1
p=0.06
COL4A1
COL4A5
p=0.09
COL7A1 HNC
p=0.02
COL7A1 MESO
p=0.01
log2 gene expression
Fresh
24 hr
Meso
HNC

E.

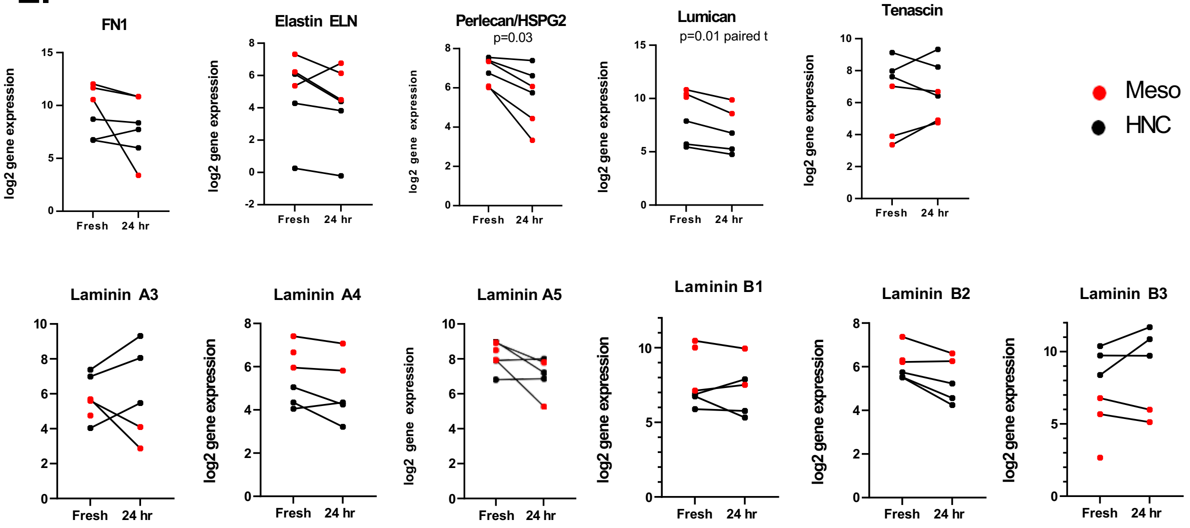

Suppl Figure 3. Gene Expression Comparisons between HNC and Mesothelioma. The mRNA expression levels (base 10) at baseline and at 24 hours for each mesothelioma (red dots) and HNC (black dots) slice are plotted for several specific genes in key categories including (A) myeloid cell attracting chemokine genes; (B) T cell and NK cell attracting chemokine genes; (C) cytokine genes; (D) collagen genes; and (E) other extracellular matrix genes. Paired t-test p values are listed when significant or borderline significant ($p< 0.05$). (HNC: n= 3; Meso: n= 3. Please note n number changes in some of the graphs).

Gene	Ave Fold change (HNC)	Ave Fold change (Meso)	Ave fold change (all)	p value (all) Wilcoxon ranked sum test
**A. Myeloid Cell Attracting Chemokines**				
CCL2 (MCP1)	0.65	4.4	2.5	0.15
CXCL8 (IL-8)	7.0	356	181	0.03
CXCL5 (ENA78)	28	346	187	0.03
CXCL3 (MIP2b)	13	28	24	0.03
CXCL2 (MIP2a)	3	15	9	0.43
CXCL1 (Groa)	0.7	4.2	2.5	0.09
**B. T cell/NK cell attracting chemokines**				
CXCL9 (MIG)	0.14	0.53	0.41	0.03
CXCL10 (IP-10)	0.14	0.5	0.37	0.03
CXCL11 (I-TAC)	0.4	0.5	0.47	0.12
CCL5 (RANTES)	0.3	0.5	0.47	0.03
**C. Cytokine Genes**				
IL1A	4.3	15.6	10	0.12
IL1B	2.0	4.6	3.3	0.21
IL6	3.7	11.4 (median)	5.3 (median	0.03
IL11	19.2	51 (median)	30 (median)	0.03
IL33	0.7	22.7	11.8	0.21
TNFa	Too low	1.55		0.25
IFNg	Too low	Too low		
IL18	0.45	0.7	0.57	0.06
**D. Collagen Genes**				
COL1A1	1.30	0.60	1.0	0.56
COL1A2	0.6	0.4	0.5	0.03
COL3A1	.6	0.5	0.50	0.06
COL4A1	0.6	1.00	0.8	0.15
COL4A2	0.7	0.9	0.8	0.06
COL4A5	0.6	0.4	0.50	0.09
COL7A1 HNC	0.5			0.02*
COL7A1 MESO		5.7		0.01*
**E. Other ECM Genes**				
FN1	1.1	.5	0.8	0.21
Elastin (ELN)	0.6	1.1	0.8	0.15
Perlecan (HSGP2)	0.7	0.5	0.6	0.03
Lumican	0.6	0.4	0.5	0.01
Tenascin	1.2	1.0	1.1	0.69
SPP1 (osteopontin)	0.6	0.4	0.5	0.03
Laminin A3	2.9	0.2	1.5	0.56
Laminin A4	0.8	0.8	0.8	0.09
Laminin A5	0.8	0.3	0.6	0.16
Laminin B1	1.1	0.8	0.9	0.43
Laminin B2	0.5	0.7	0.6	0.06

Suppl Table 3. Chemokine, Cytokine, and extracellular Matrix mRNA Expression Changes

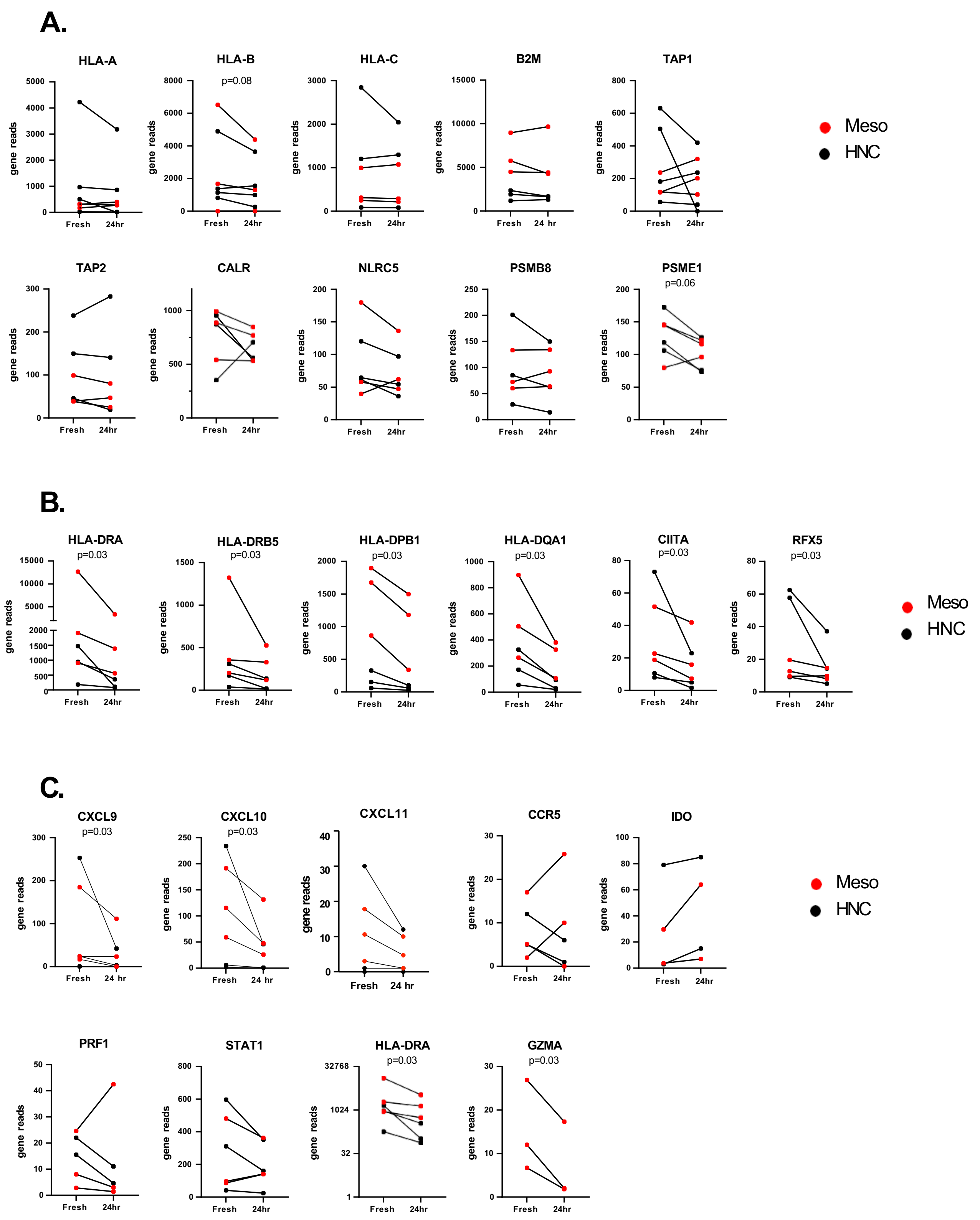
A.
HLA-A
HLA-B
p=0.08
HLA-C
B2M
TAP1
TAP2
CALR
NLRC5
PSMB8
PSME1
p=0.06
gene reads
Fresh
24hr
Meso
HNC
B.
HLA-DRA
p=0.03
HLA-DRB5
p=0.03
HLA-DPB1
p=0.03
HLA-DQA1
p=0.03
CIITA
p=0.03
RFX5
p=0.03
Meso
HNC
C.
CXCL9
p=0.03
CXCL10
p=0.03
CXCL11
CCR5
IDO
PRF1
STAT1
HLA-DRA
p=0.03
GZMA
p=0.03
Meso
HNC

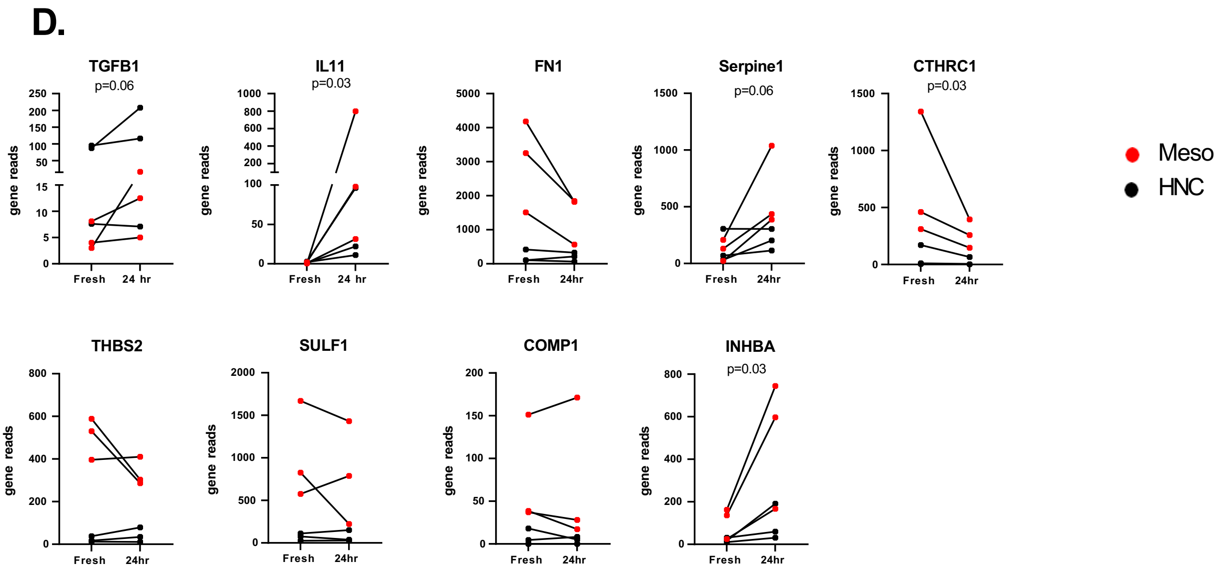

Suppl Figure 4. Gene Expression Comparisons between HNC and Mesothelioma. The mRNA expression levels (base 10) at baseline and at 24 hours for each Meso (red dots) and HNC (black dots) slice are plotted for a number of specific genes in key categories including: (A) HLA Class 1 and antigen-presenting machinery genes; (B) HLA Class 2 Genes; (C) Interferon-γ induced Genes; and D) TGFβ induced Genes. Paired t-test p values are listed when significant or borderline significant ($p < 0.05$). (HNC: n= 3; Meso: n= 3. Please note n number changes in some of the graphs).

Gene	Ave Fold change (HNC)	Ave Fold change (Meso)	Ave fold change (all)	p value (all) Wilcoxon ranked sum test
**A. HLA and APM Genes**				
HLA-A	0.79	1.24	1.01	0.29
HLA-B	0.74	0.77	0.75	0.08
HLA-C	0.91	0.95	0.93	0.84
B2M	0.9	0.94	0.92	0.56
TAP1	0.89	1.32	1.11	0.81
TAP2	0.85	0.88	0.87	0.56
NLRC5	0.75	1.05	0.90	0.16
CALR	1.07	0.90	0.99	0.31
PSMB8	0.66	1.11	0.88	0.58
PSME1	0.69	0.95	0.82	0.06
**B. HLA Class 2 Genes**				
HLA-DRA	0.29	0.54	0.41	0.03
HLA-DRB5	0.31	0.64	0.47	0.03
HLA-DMB	0.24	0.39	0.32	0.03
HLA-DPB1	0.38	0.63	0.50	0.03
HLA-DQA1	0.28	0.49	0.38	0.03
CIITA	0.36	0.63	0.50	0.03
RFX5	0.47	0.80	0.64	0.06
**C. Interferon-γ Stimulated Genes**				
CXCL9 (MIG)	0.14	0.53	0.41	0.03
CXCL10 (IP-10)	0.14	0.5	0.37	0.03
CXCL11 (I-TAC)	0.4	0.5	0.47	0.12
CCR5	0.33	2.5	1.64	0.81
IDO	1.08	3.1	2.61	0.13
PRF1	0.40	0.9	0.67	0.63
STAT1	0.57	1.3	0.93	0.31
HLA-DRA	0.29	0.5	0.41	0.03
GZMA	Too low	0.32		0.03
**D. TGF-β Stimulated Genes**				
TGFB1	1.51	2.76	2.133	0.06
IL-11	19.7	51 (median)	32 (median)	0.03
FN1	1.12	0.46	0.79	0.16
COL1A1	1.34	0.64	0.99	0.06
SERPINE1	3.02	8.29	5.64	0.06
CTHRC1	0.48	0.44	0.46	0.03
THBS2	1.67	0.70	1.18	0.84
SULF1	0.98	0.83	0.91	0.69
COMP	1.09	0.77	0.93	0.62
INHBA	5.41	5,21	5.31	0.03

Suppl Table 4. HLA, Interferon-g and TGF-b mRNA Expression Changes

A.

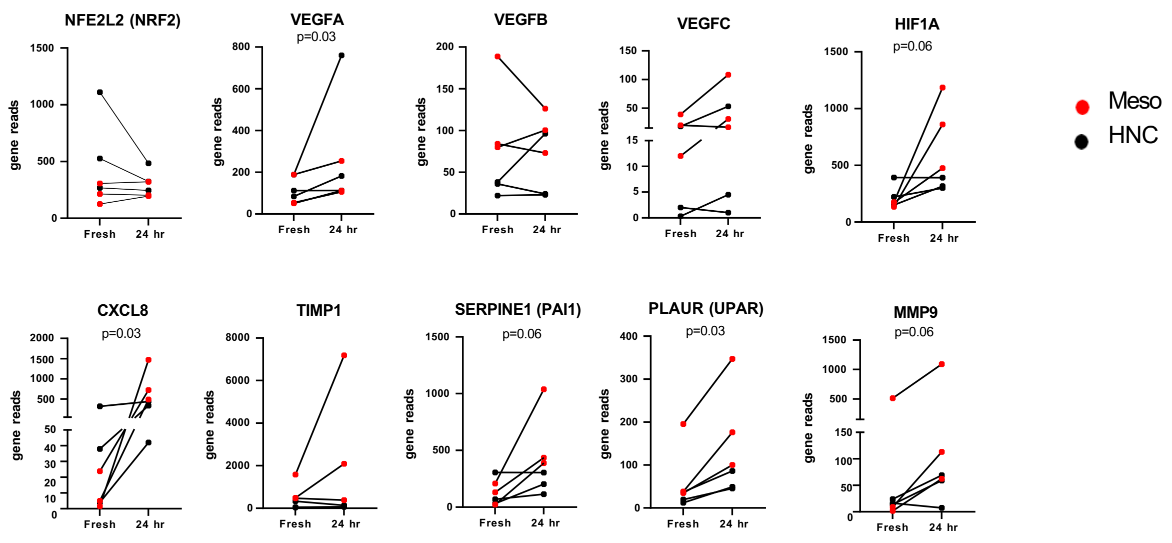
NFE2L2 (NRF2)
VEGFA
p=0.03
VEGFB
VEGFC
HIF1A
p=0.06
CXCL8
p=0.03
TIMP1
SERPINE1 (PAI1)
p=0.06
PLAUR (UPAR)
p=0.03
MMP9
p=0.06
gene reads
Fresh
24 hr
Meso
HNC

B.

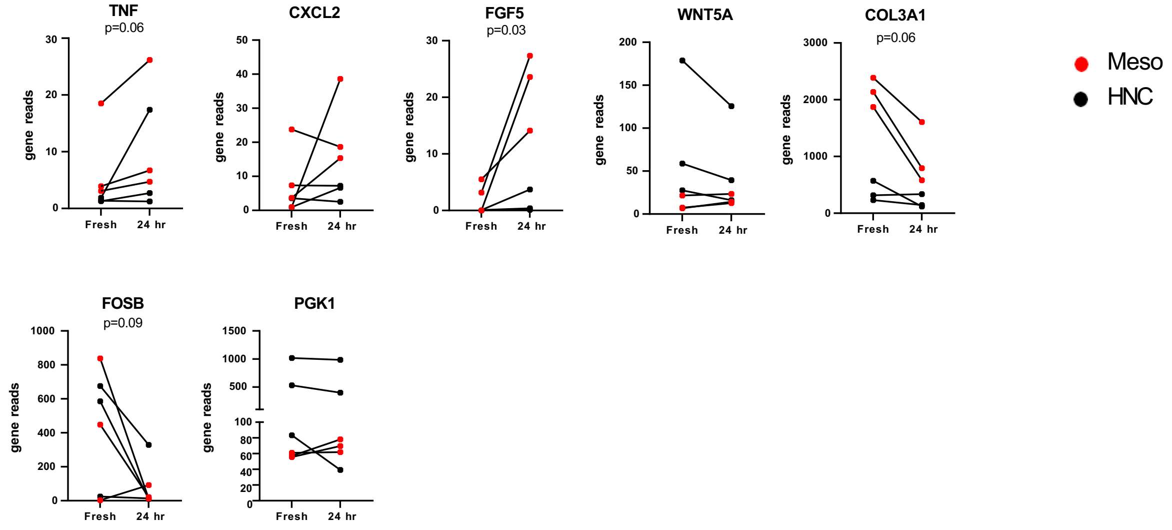
TNF
p=0.06
CXCL2
FGF5
p=0.03
WNT5A
COL3A1
p=0.06
FOSB
p=0.09
PGK1
gene reads
Fresh
24 hr
Meso
HNC

C.

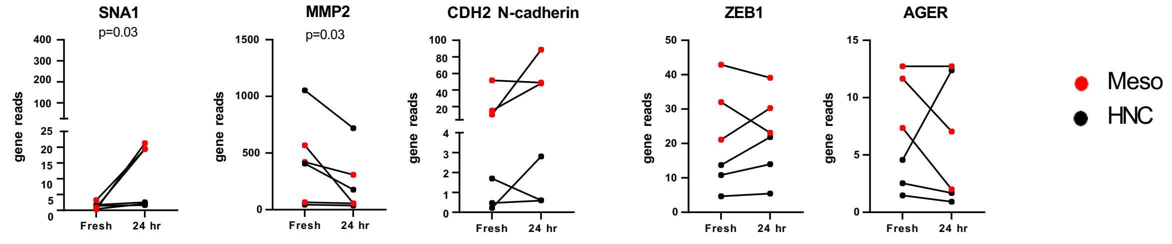
SNA1
p=0.03
MMP2
p=0.03
CDH2 N-cadherin
ZEB1
AGER
gene reads
Fresh
24 hr
Meso
HNC

D.

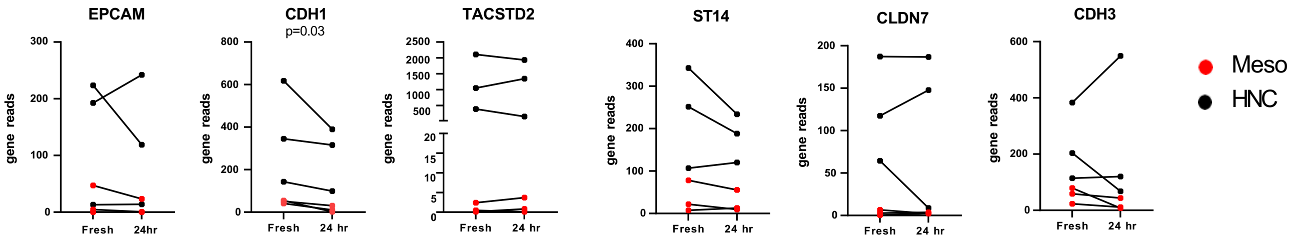

E.

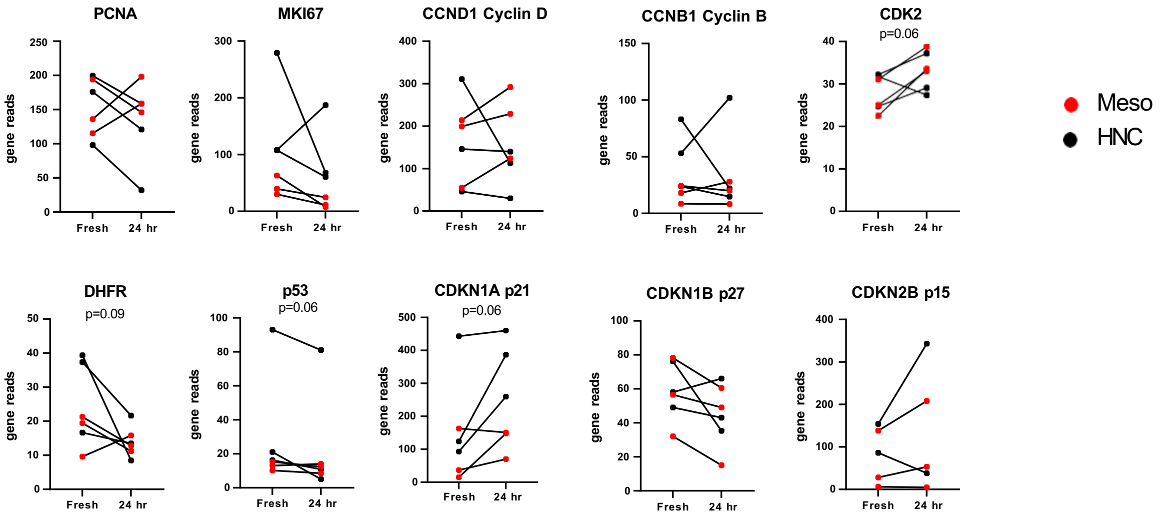

Suppl Figure 5. Gene Expression Comparisons between HNC and Mesothelioma. The mRNA expression levels (base 10) levels at baseline and at 24 hours for each Meso (red dots) and HNC (black dots) slice are plotted for a number of specific genes in key categories including: Hypoxia-induced genes; (B) Wound-healing signature genes; (C) EMT- mesenchymal genes; (D) EMT- epithelial genes; (E) Proliferation and cell cycle genes. Paired t-test p values are listed when significant or borderline significant ($p < 0.05$). (HNC: n= 3; Meso: n= 3)

Gene	Ave Fold change (HNC)	Ave Fold change (Meso)	Ave fold change (all)	p value (all) Wilcoxon ranked sum test
**A. Hypoxia-Related Genes**				
NFE2L2 (NRF2)	0.7	1.2	0.9	0.43
VEGFA	2.4	1.8	2.1	0.03
VEGFB	1.4	0.9	1.2	0.99
VEGFC	6.7	2.1	4.4	0.15
HIF1A	1.5	5.3	3.4	0.06
CXCL8	7.0	356	182	0.03
TIMP1	1.1	3.2	2.1	0.56
SERPINE1 (PAI1)	3.0	8.3	5.7	0.06
PLAUR (UPAR)	2.9	3.1	3.0	0.03
MMP9	2.5	26.5	14.5	0.06
**B. Wound Healing Signature Genes**				
TNF	4.1	1.6	2.8	0.06
CXCL2	0.98 (median)	4.1 (median)	2.6 (median)	0.43
FGF5	5.1 (median)	1.5 (median)	1.6 (median)	0.03
WNT5A	0.7	1.6	1.1	0.43
COL3A1	0.6	0.5	0.6	0.06
FOSB	0.4	9.7	5.0	0.09
PGK1	0.7	1.2	1.0	0.43
**C. EMT genes – Mesenchymal Genes**				
SNAI1	2.5	31.6	17	0.03
SNAI2	1.7	0.9	1.3	0.68
ZEB1	1.4	1.0	1.2	0.68
ZEB2	1.1	1.5	1.3	0.15
FN1	1.1	0.5	0.8	0.15
MMP2	0.6	0.6	0.6	0.03
COL1A1	1.3	0.7	1.0	0.68
AGER	1.3	0.6	1.0	0.62
CDH2 (N-cadherin)	4.8	4.2	4.5	0.43
**D. EMT genes – Epithelial genes**				
EPCAM	1.0	0.2	0.6	0.56
CDH1	0.7	0.3	0.5	0.03
CLDN7	0.8	2.4	1.6	0.71
CDH3 (P cadherin)	0.9	0.4	0.7	0.56
CTNND1	1.2	0.9	1.0	0.99
CTNNA1	1.4	1.2	1.3	0.99
TACSTD2	0.9	2.7	1.8	0.99
ST14	0.9	0.9	0.9	0.21

Suppl Table 5. Hypoxia-Related, Wound Healing, and EMT mRNA Expression Changes

Gene	Ave Fold change (HNC)	Ave Fold change (Meso)	Ave fold change (all)	p value (all) Wilcoxon ranked sum test
**A. Proliferation Genes**				
PCNA	0.60	1.19	0.90	0.56
MKI67	0.84	0.37	0.61	0.31
CCND1 (cyclin D1)	0.66	1.58	1.12	0.84
CCNB1 (cyclin B1)	0.93	1.11	1.02	0.84
CDK2 (cyclin dependent kinase 2)	1.06	1.35	1.21	0.06
RB1	1.26	1.56	1.41	0.21
**B. Cell Cycle Inhibitor Genes**				
TP53	0.60	0.92	0.76	0.06
CDKN1A-p21	2.32	4.17	3.24	0.06
CDKN1B-p27	0.83	0.70	0.77	0.15
CDKN2B-p15	1.87	1.05	1.38	0.43

Suppl Table 6. Proliferation and Cell Cycle Inhibitor mRNA Expression Changes

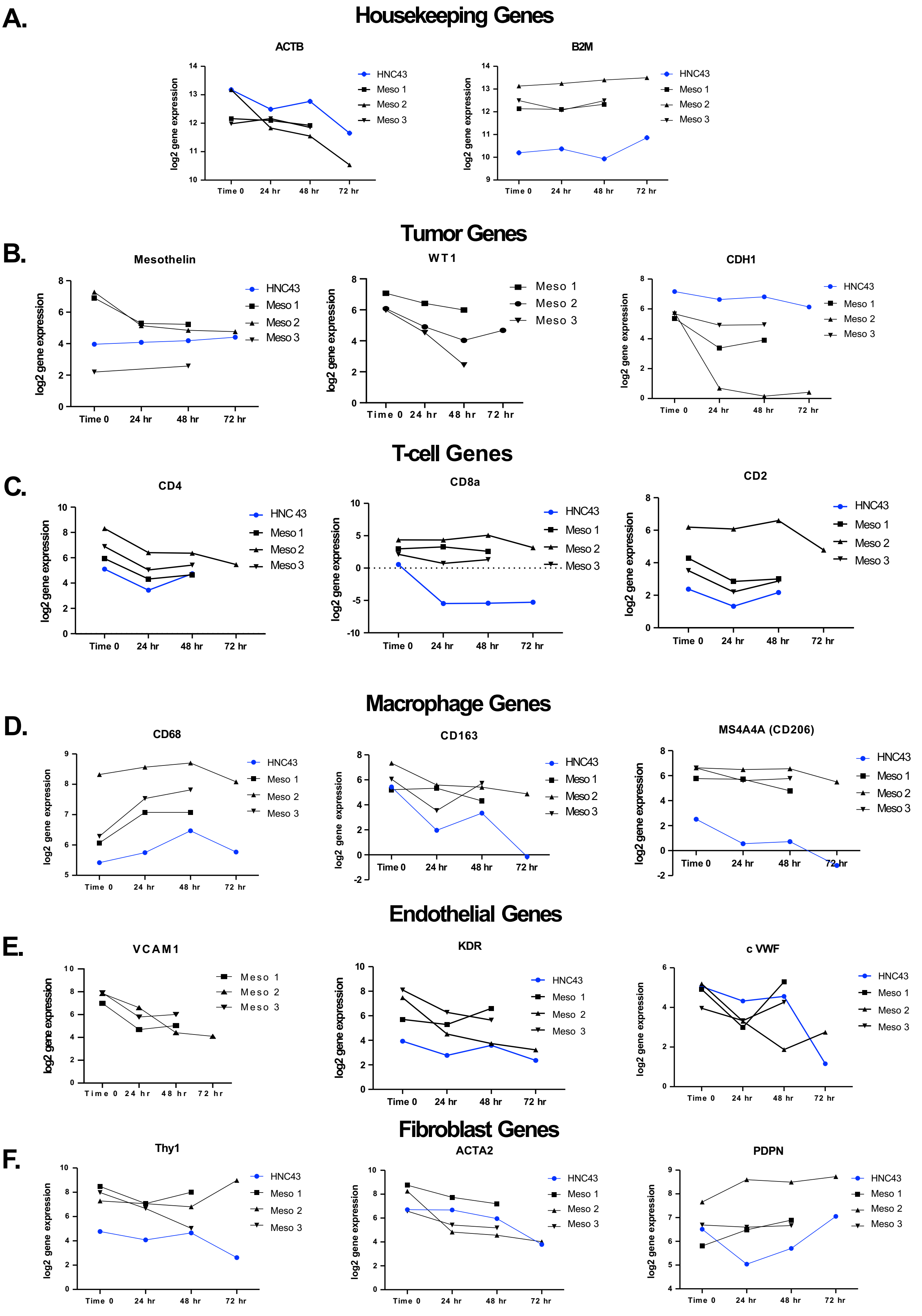
A.
Housekeeping Genes
ACTB
B2M
B.
Tumor Genes
Mesothelin
WT1
CDH1
C.
T-cell Genes
CD4
CD8a
CD2
D.
Macrophage Genes
CD68
CD163
MS4A4A (CD206)
E.
Endothelial Genes
VCAM1
KDR
c VWF
F.
Fibroblast Genes
Thy1
ACTA2
PDPN
log2 gene expression
Time 0
24 hr
48 hr
72 hr
HNC43
HNC 43
Meso 1
Meso 2
Meso 3

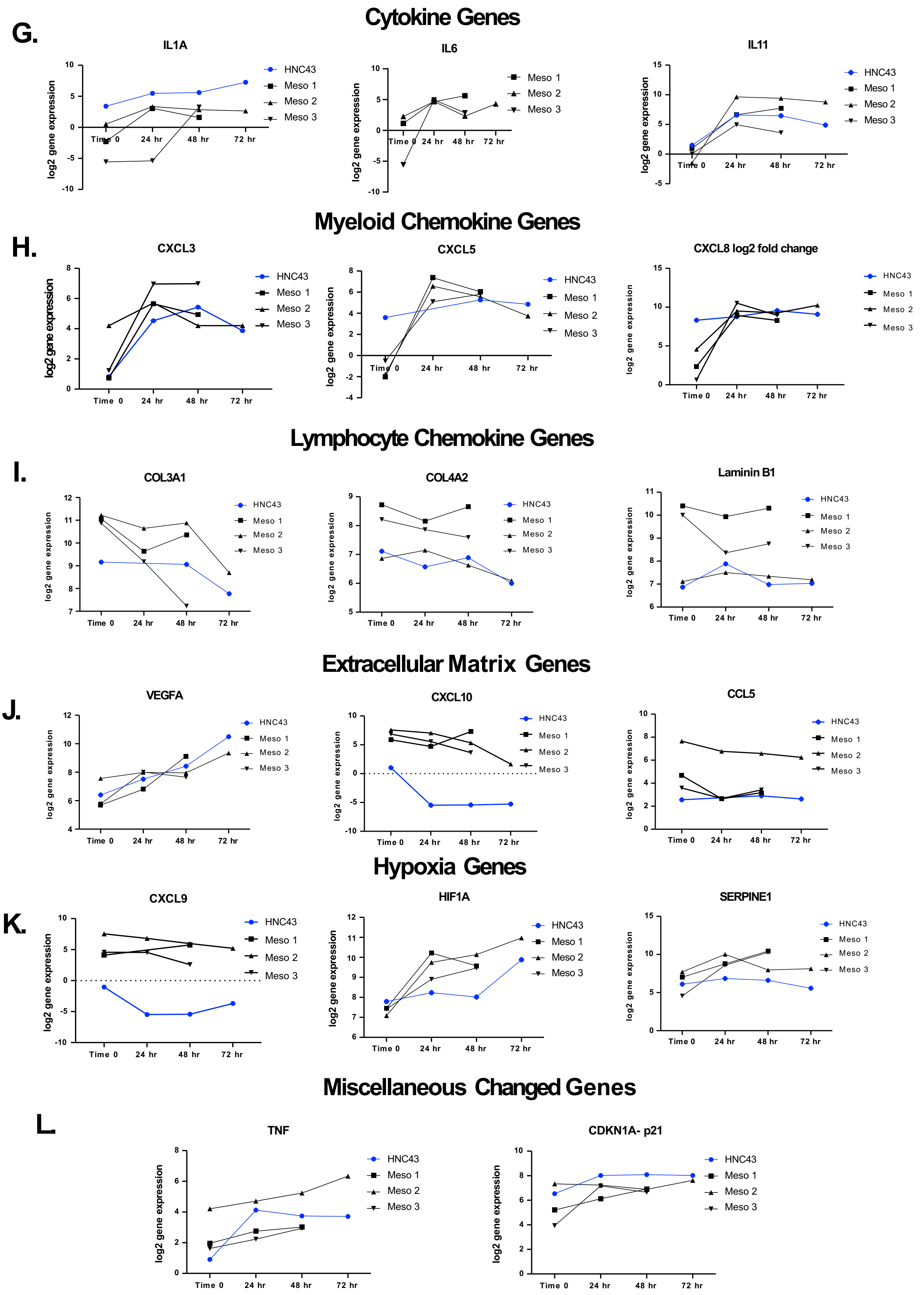

Cytokine Genes
G.
IL1A
IL6
IL11
log2 gene expression
Time 0
24 hr
48 hr
72 hr
HNC43
Meso 1
Meso 2
Meso 3
Myeloid Chemokine Genes
H.
CXCL3
CXCL5
CXCL8 log2 fold change
Lymphocyte Chemokine Genes
I.
COL3A1
COL4A2
Laminin B1
Extracellular Matrix Genes
J.
VEGFA
CXCL10
CCL5
Hypoxia Genes
K.
CXCL9
HIF1A
SERPINE1
Miscellaneous Changed Genes
L.
TNF
CDKN1A- p21

Suppl Figure 6. Gene expression changes over time. The log2 gene expression values in PCTS from 4 different tumors (Meso n= 3; HNC n= 1)) were measured at baseline (time 0), and at 24 48 and 72 hrs in culture. Data are plotted for a number of specific genes in key categories including (A) housekeeping genes, (B) tumor-selective genes, (C) T cell genes, (D) macrophage genes, (E) endothelial genes, (F) fibroblast genes, (G) cytokine genes, (H) myeloid cell attracting chemokine genes, (I) lymphocyte attracting chemokine genes, (J) extracellular matrix genes, (K) hypoxia-related genes, (L) miscellaneous genes. (HNC: n= 3; Meso: n= 3)
